# Protein Glutathionylation and Glutaredoxin: Role in Neurodegenerative Diseases

**DOI:** 10.3390/antiox11122334

**Published:** 2022-11-25

**Authors:** Haseena P. A., Latha Diwakar, Vijayalakshmi Ravindranath

**Affiliations:** 1 Centre for Brain Research, Indian Institute of Science, Bangalore 560012, India; 2 Manipal Academy of Higher Education (MAHE), Manipal 576104, India

**Keywords:** oxidative stress, glutathione, glutaredoxin, protein thiol, ischemia, Parkinson’s disease, Alzheimer’s disease

## Abstract

Oxidative stress has been implicated in the pathogenesis and progression of many neurodegenerative disorders including Parkinson’s disease and Alzheimer’s disease. One of the major enzyme systems involved in the defense against reactive oxygen species are the tripeptide glutathione and oxidoreductase glutaredoxin. Glutathione and glutaredoxin system are very important in the brain because of the oxidative modification of protein thiols to protein glutathione mixed disulfides with the concomitant formation of oxidized glutathione during oxidative stress. Formation of Pr-SSG acts as a sink in the brain and is reduced back to protein thiols during recovery, thus restoring protein functions. This is unlike in the liver, which has a high turnover of glutathione, and formation of Pr-SSG is very minimal as liver is able to quickly quench the prooxidant species. Given the important role glutathione and glutaredoxin play in the brain, both in normal and pathologic states, it is necessary to study ways to augment the system to help maintain the protein thiol status. This review details the importance of glutathione and glutaredoxin systems in several neurodegenerative disorders and emphasizes the potential augmentation of this system as a target to effectively protect the brain during aging.

## 1. Introduction

The brain is highly sensitive to oxidative stress due to its high oxygen consumption, abundance of unsaturated fatty acids which are prone to oxidation, and low antioxidant levels. It is a metabolically active and a high energy demanding organ that relies heavily on mitochondria for its energy needs [1,2]. Majority of oxygen consumed by mitochondria during oxidative phosphorylation is coupled to ATP synthesis while ~4% contributes to the generation of superoxides which are further metabolized to reactive oxygen species (ROS) [3]. ROS modify proteins causing functional and structural damage to biomolecules. Prolonged exposure to ROS also damages DNA, mitochondrial membranes, and lipids [4], impairing its metabolic functions including synthesis of ATP, fatty acid oxidation and metabolism of essential biomolecules [3].

Oxidative stress results from the excess accumulation of reactive oxygen species, such as superoxides, hydrogen peroxides, hydroxyl radicals and nitric oxide when they are not effectively counteracted by antioxidants [5]. Oxidative stress and mitochondrial dysfunction play a major role in the pathogenesis and progression of neurodegenerative disorders [6,7]. Redox enzymes as well as antioxidants are present in lower concentration in the brain compared with other organs [7,8] and are distributed differentially in brain regions. The redox defense system is critically important in the brain as the overproduction of ROS leads to neurodegeneration. 

Reactive oxygen species are detoxified through a set of enzymes including superoxide dismutase, catalase and glutathione peroxidase. Super oxide dismutase (SOD) catalyzes the conversion of superoxide molecules to hydrogen peroxide [9]. It is present in two forms namely Mn-SOD and Cu,Zn-SOD which are located in mitochondria and cytosol respectively [10]. Hydrogen peroxide is formed and is thus reduced to water by catalase present in peroxisome [11] and by glutathione peroxidase [12]. Peroxiredoxin, a cysteine-dependent peroxidase is also involved in reducing hydrogen peroxide, lipid hydroperoxides and peroxynitrite. The inability of these enzymes to metabolize the excessive hydrogen peroxide results in the production of hydroxyl radicals. The hydroxyl radicals are potent prooxidants causing irreversible damage to major macromolecules of the cell such as DNA, lipids and proteins [13].

**Figure 1:**
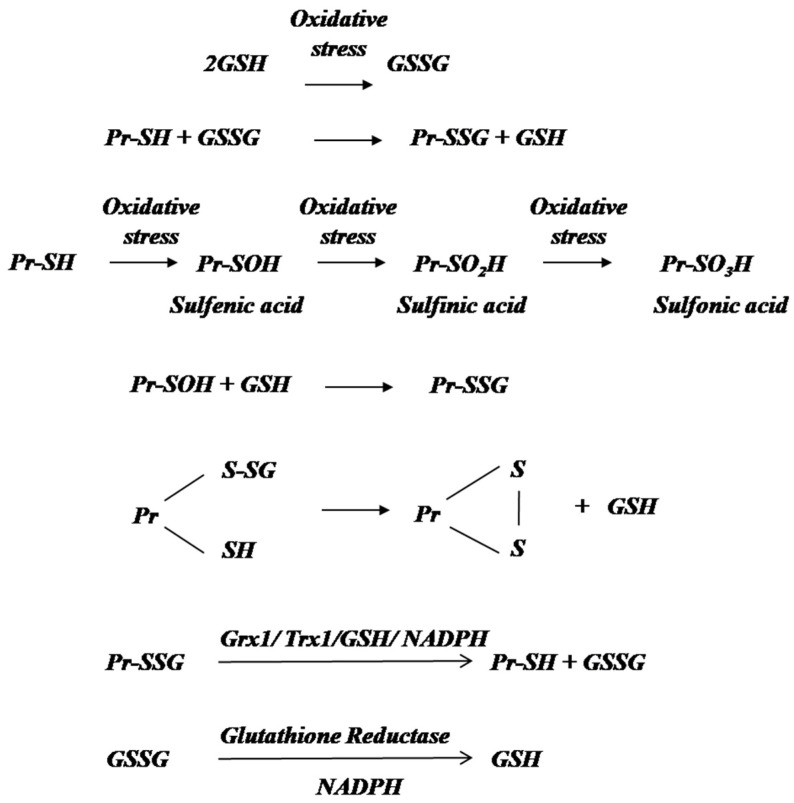
Protein thiol modification during oxidative stress. During oxidative stress, GSH is oxidized to GSSG, which promotes the oxidative modification of protein thiols (Pr-SH) to protein glutathione mixed disulfides (Pr-SSG). Protein thiols can be directly oxidized to organosulfur oxoacids sulfenic (Pr-SOH), sulfinic (Pr-SO2H) and sulfonic acid (Pr-So3H). Sulfenic acid can be glutathionylated to prevent its further oxidation to irreversible sulfonic acids. Pr-SSG can undergo further modifications to form protein mixed disulfides. Pr-SSGs are reduced back to protein thiols by Grx1 or Trx1 utilizing the reducing equivalent of NADPH. GSSG formed in this reaction is effectively reduced to GSH by glutathione reductase using the reducing equivalent of NADPH.

Glutathione (GSH), the tripeptide in the form of γ-glutamyl cysteinyl glycine, is a major thiol antioxidant responsible for the maintenance of protein thiol status in the cells. During the detoxification of ROS, glutathione peroxidase (GPx) oxidizes GSH to GSSG. The oxidized form GSSG connected by an intermolecular disulfide bond is reduced back by glutathione reductase (GR) utilizing the reducing equivalents of NADPH [14] (Figure 1). The ratio of GSH/GSSG can effectively help determine the ongoing oxidative stress in tissues [15,16,17].

One of the major changes that occur even under low levels of oxidative stress is the changes in protein thiols leading to their successive oxidation to disulfides, sulfenic, sulfinic and sulfonic acid (Figure 1). Sulfenic and sulfinic acids formed are reduced to thiols by enzymes such as glutaredoxin and sulferedoxin, thus preventing the formation of sulfonic acid that causes irreversible damage to the protein [18]. In addition, protein thiols can be directly oxidized to protein glutathione mixed disulfides (Pr-SS-Pr) in the presence of GSH (Figure 1). Glutaredoxin has a dual role; in the glutathionylation of sulfenylated proteins to prevent further oxidation and, most importantly in the reduction PrSSG to PrSH thus bringing back proteins to their active thiol utilizing NADPH. Oxidoreductases such as glutaredoxin (Grx) and thioredoxin (Trx) play an important role in maintaining protein thiol homeostasis in cells [19]. Although functionally similar, Grx is more versatile than Trx in terms of substrate reduction and reaction mechanisms [20].

Glutaredoxins (Grx) are evolutionarily conserved GSH-dependent oxidoreductase involved in the regulation of several cellular processes [21]. Grx1 regulates the S-glutathionylation of proteins particularly those involved in cell death and cell survival pathways [22]. It catalyzes the reduction of disulfides and mixed disulfides to protein thiols by a process called deglutathionylation. Oxidized glutathione is reduced by glutathione reductase utilizing the reducing equivalent of NADPH leading to the formation of GSSG. Glutaredoxins utilize the di-thiol mechanisms to remove the internal disulfide bridges in proteins [23] and mono-thiol mechanisms to control S-glutathionylation. Multiple forms of glutaredoxin are expressed ubiquitously. Of these, Grx1 is cytosolic, Grx2 is mitochondrial [24] and Grx3 [25] is located in the nuclear compartment. Grx1 is the most predominant form present in the brain.

Oxidative stress and a dysfunctional redox system are implicated in the initiation and progression of many neurodegenerative disorders such as Alzheimer’s disease (AD) and Parkinson’s disease (PD) [26]. Selected neuronal populations show higher susceptibility toward oxidative stress [27]. Dopaminergic neurons with metal content are particularly vulnerable to oxidative damage [28]. Perturbation in protein thiol homeostasis is well documented in AD as well as in PD brains. Thus, the review will focus on protein thiol oxidation in the brain and the system involved in the maintenance of thiol homeostasis during neurodegeneration.

### 1.1. Brain Glutathione

GSH has critical roles in maintaining the brain redox status and protecting brain cells from oxidative damage [29]. It is the most abundant non-protein thiol that can directly scavenge free radicals to detoxify oxidants. GSH exists in two forms in the cell, reduced GSH which undergoes oxidation during ROS scavenging and serves as a substrate for thiol oxidoreductase enzymes, and the oxidized form, GSSG. GSH synthesis is catalyzed by a two-step enzymatic reaction: γ-glutamylcysteine synthetase (γ-GCL/GCS) combines glutamate and cysteine to generate γGlu-Cys which is merged with glycine by GSH synthetase (GS) to form tripeptide, GSH. The rate of GSH synthesis depends on the activity of the rate limiting enzyme γ-GCL and the availability of amino acid cysteine [30]. The cytosolic and mitochondrial pool of GSH is present in cells. Mitochondrial GSH concentration is 1–2% of the cytosolic GSH and is maintained by the uptake of cytosolic GSH. GSH synthesis is an energy-driven process and any mitochondrial dysfunction that reduces ATP synthesis affects the total GSH pool [31]. Moreover, GSH turnover in the brain is slower, ~72 h compared with the liver which has a turnover time of 4 h.

**Figure 2:**
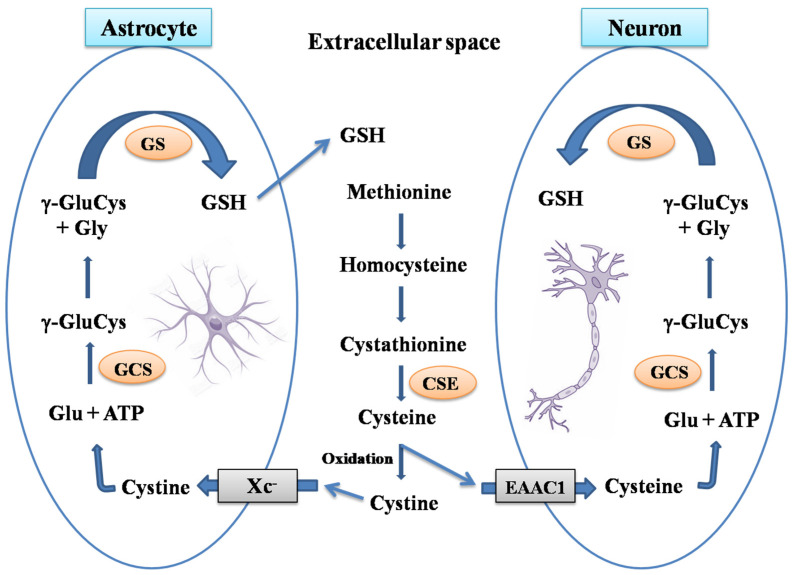
GSH synthesis in astrocytes and neurons. The Transsulfuration pathway synthesizes homocysteine from the dietary amino acid methionine to produce cystathionine. Further, cystathionine-γ-lyase (CSE) converts cystathionine into cysteine in extracellular to neurons and astrocytes. Neurons utilize extracellular cysteine for GSH synthesis, unlike astrocytes which uses the oxidized form cystine. Neurons uses excitatory amino acid transporter (EAATs) to take up cysteine, while a cystine/glutamate transporter (Xc-) mediates the transport of cystine in astrocytes. γ-Glutamylcysteine synthetase (GCS), rate limiting enzyme in GSH synthesis catalyzes the formation of γ-glutamylcysteine dipeptide utilizing ATP. γ-GluCys is then combined with glycine to form GSH by glutathione synthetase (GS) in both neurons and astrocytes. GSH formed in astrocytes is released into extracellular space where it is cleaved into individual aminoacids by a set of enzymes.

Astrocytes have higher GSH concentration compared to neurons, and both have a differential preference for the precursors of GSH synthesis. Secretion of GSH into the extracellular space by astrocytes provides GSH precursors to neurons [32] (Figure 1). In addition, cysteine transport to the cell also occurs differently in neurons and astrocytes. Neurons use excitatory aminoacid transporter (EAATs) to take up cysteine from extracellular space, while cystine/glutamate transporter (Xc-) mediates transport in astrocytes (Figure 2). Of the five EAATs known to exist, excitatory amino acid carrier 1 (EAAC1) is involved in the neuronal uptake of cysteine [33]. Neurons use extracellular cysteine for GSH synthesis and cannot use oxidized form cystine, unlike astrocytes. Transsulfuration pathways provide intracellular cysteine for GSH synthesis. The majority of GSH synthesized in the liver utilizes the cysteine formed from transsulfuration. The activity of cystathionine-γ-lyase (the rate-limiting enzyme in transsulfuration pathway) in the brain is approximately 1% of the corresponding liver activity [34]. However, astrocytes also depend on transsulfuration pathways for intracellular cysteine availability, while it is nearly absent in neurons. Thus, the availability of cysteine is very critical to neurons that exclusively depend on the extracellular cysteine for GSH synthesis [35].

Replenishing GSH in the brain could be a promising treatment strategy for neurodegenerative disorders. GSH does not cross blood-brain-barrier and its systemic administration is not effective to increase the concentration in the brain as most of the GSH are metabolized in blood. Several studies have investigated the effect of GSH precursors in a mouse model in restoring GSH. Treatment with GSH precursors has been shown to protect from Aβ induced neurotoxicity [36] and cognitive impairment in AD mouse models. N-acetyl-cysteine, a membrane-permeable cysteine precursor with a potent antioxidant activity helps to reduce oxidative stress and cognitive decline promoting neuronal survival [37]. NAC also promotes the reduction of GSSG to GSH. Oral administration of γ-glutamylcysteine (γ-GC) to APP/PS1 mice increased the GSH content and ratio of GSH/GSSG restoring spatial memory deficit. In vitro treatment of γ-GC attenuate the BSO induced GSH reduction and protects from oligomeric Aβ [38]. Recently, a drug delivery system using ultrasound combined with microbubbles containing anti-miR- 96-5p successfully increased the EAAC1 and GSH levels in mice hippocampus. MicroRNA miR-96-5p increases the levels of GTRAP3-18, an inhibitor of cysteine transporter EAAC1 [39]. Although several studies reported beneficial effects of restoring GSH in a mouse model, they have shown minimal benefits to patients. Further studies need to be performed to effectively augment the redox system to combat oxidative stress-mediated dysfunctions in the brain.

### 1.2. Glutaredoxin

**Figure 3:**
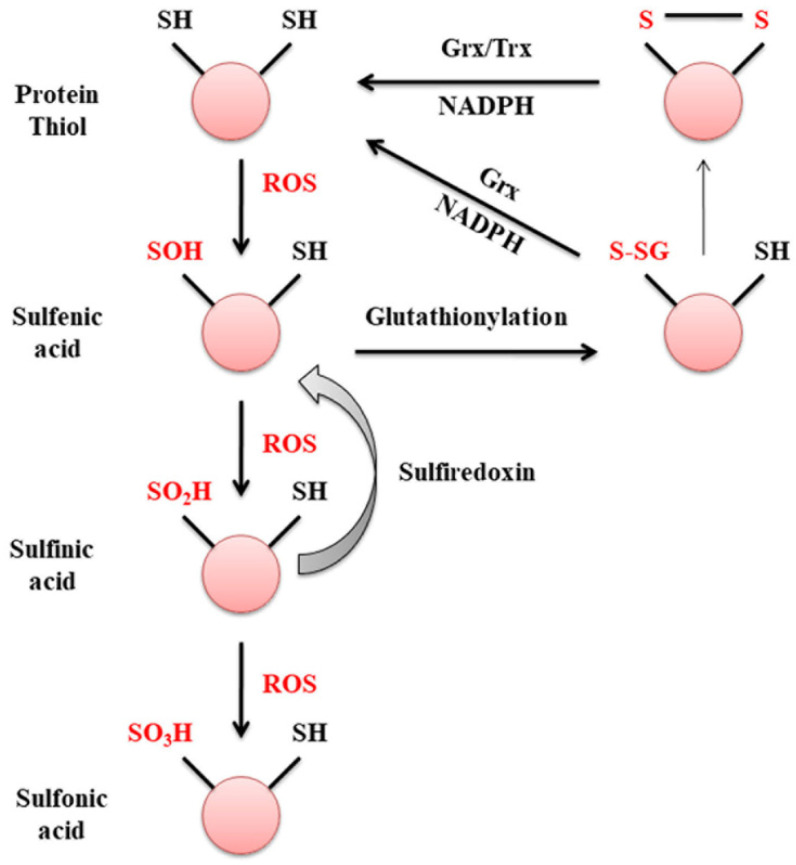
Oxidation of protein thiols to sulfonic acids. During oxidative stress, ROS such as hydrogen peroxide can catalyze the two-electron oxidation of sulfur present in amino acid cysteine. Protein thiols are oxidized to sulfenic acid, which can react with GSH to form protein disulfide preventing further oxidation. Pr-SSG formed is reduced back to active thiols by glutaredoxin or thioredoxin using reducing equivalent NADPH. Sulfinic acid formed by subsequent oxidation of sulfenic acid is still reversible by the reductase enzyme, sulferedoxin. Further oxidation of sulfinic acid results in an irreversible formof sulfonic acid, which targets the protein for degradation.

Glutaredoxins are ubiquitously expressed thiol oxidoreductases that regulate protein thiol homeostasis by reversing protein S-glutathionylation using GSH as a substrate. Grx1 regulates proteins involved in cell survival and death including Akt, cjun, ion transporters, NFκB, intracellular signaling molecule Ras and transcription factor nuclear factor-1 (NF1) [22]. A major function of Grx1 in the cell is to maintain protein thiol status (Pr-SH) or sulfhydryl homeostasis during oxidative damage [40]. Protein thiols undergo oxidation to sulfenic acid, an intermediate in the oxidation pathway that readily reacts to form intramolecular disulfide bonds. Sulfenylated cysteines can be glutathionylated by glutaredoxins and reduced back to active protein thiols. Further oxidation of sulfenic acid generates sulfinic acid which can be reduced by sulfiredoxin (Figure 3). The formation of sulfonic acid is irreversible, where it inhibits protein functions and targets them for degradation. There are no known enzymes present to catalyze the reduction of sulfonic acids due to the low pH needed for its reduction [18]. Thus, Grx1 induces the formation of reversible protein S-glutathionylation to protect proteins from irreversible modifications by ROS.

The glutaredoxin-mediated reduction of protein mixed disulfides to protein thiols is critical in the brain where glutathionylation of protein is more prominent. In the brain, under conditions of oxidative stress such as that seen during reperfusion following ischemia or in animal models of MPTP, there is a predominant formation of Pr-SS-Pr. During recovery following these insults, Pr-SSG is reduced back to Pr-SH by Grx1, thus restoring protein function.

Glutaredoxin was first purified by Mieyal et al. from human erythrocytes as thioltransferase specific for GSH containing mixed disulfides [41] and cloned in Escherichia coli [42]. In rat brains, a constitutive expression of glutaredoxin exhibits regional and cellular variability in its localization. Glutaredoxin activity is detected higher in the hippocampus where they could recover more rapidly from oxidative damage compared to other regions with less activity in the cerebellum and striatum [43]. A similar study carried out using human autopsy brain samples detected higher glutaredoxin activity in the hippocampus and cerebellum. Glutaredoxins are localized predominantly in neurons in the cerebral cortex and hippocampus, purkinje and granule cell layers of the cerebellum, and granular cell layers of the dentate gyrus in the human brain [44].

### 1.3. Glutaredoxin and Mitochondrial Dysfunction

Maintaining protein thiol homeostasis is important for mitochondrial functions. Loss of GSH and formation of Pr-SSG in the brain contributes to various dysfunctions including loss of activity of mitochondrial enzymes, structural damage to mitochondrial membranes and deregulation of cell survival pathways. Mitochondria are prone to oxidative damage as ROS generated during oxidative phosphorylation can in turn releases more reactive oxygen species that can damage the macromolecules essential for its normal function. The percentage of total cell GSH present in mitochondria is 10–15% [45].

The response of brain mitochondria to oxidative stress is very different from the liver mitochondria. In the brain, the GSH lost during oxidative stress is recovered as Pr-SSG and only less than 5% of GSH is recovered as GSSG [46]. However, in the liver, most of the GSH lost is recovered as GSSG, and a reduced state of protein thiols are maintained. The excess GSSG formed is effluxed out of the cell to prevent oxidative modification of proteins [47].

Mitochondrial enzymes are particularly vulnerable to oxidative damage, complex Ⅰ being the most affected as it increases the oxidation of the GSH pool. Protein thiol groups on 75 and 51 KDa subunits of complex Ⅰ undergo glutathionylation and forms mixed disulfide with GSH [48] (Figure 2). Thiol modifications on complex Ⅰ inhibit its activity and increase ROS production that can be reversed by either Grx1 or the disulfide reducing agent dithiothreitol [49]. Downregulation of Grx1 in the brain regions of Swiss albino mice results in a significant loss of complex Ⅰ activity signifying the role of glutaredoxins in mitochondrial function [50]. ROS-mediated inhibition of activity is specific to complex Ⅰ while other complexes in the electron transport chain remain unaffected.

Glutaredoxin 2 (Grx2) was first identified and cloned by Holmgren et al., which is 34% identical to cytosolic Grx1 [51]. It has three isoforms: Grx2a is localized to mitochondria, and Grx2b and Grx2c are both localized to the nucleus and cytosol [52]. Grx2a, a mitochondrial isoform of glutaredoxin regulates the glutathionylation of mitochondrial enzymes. Constitutive expression of Grx2 is observed in mouse and human brains and is localized to neurons and glia cells including the neurons of substantia nigra. Grx2 expression is transiently upregulated in MPTP-treated mice and a partial loss in complex Ⅰ activity due to Grx2 downregulation [53]. Theoverexpression of Grx2 prevents apoptosis by inhibiting cytochrome c release and caspase activation [54] and could ameliorate the toxic effect of MPP^+^ in mitochondria [53].

Further, overexpression of Grx2 attenuatesthe mutant superoxide dismutase (SOD1) mediated degeneration of motor neurons. MutSOD1 accumulates in the mitochondria of motor neurons, and impairs respiratory complexes and ATP production. Grx2 is required for normal mitochondrial function and viability, whereas overexpression of Grx1 is shown to be beneficial in the cytosol and does not preserve mitochondrial dynamics or apoptosis induced by mutSOD1 [55].

**Figure 4:**
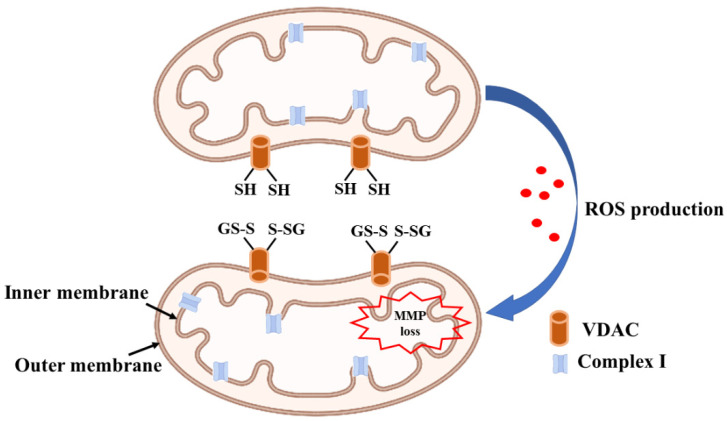
Oxidative stress leads to complex Ⅰ inhibition and mitochondrial membrane potential loss. Protein thiols present in VDAC undergo oxidation leading to increased permeability of transition pore and loss of mitochondrial membrane potential. MMP loss facilitates the release of apoptotic factors such as cytochrome c promoting cell death.

Increased Pr-SSG formation due to loss of Grx1 activity in the cytosol is known to impair mitochondrial dynamics in the brain. Pr-SSG formed by the oxidation of sulfhydryl groups of cysteine increases the permeability of the inner mitochondrial membrane and opens upa mitochondrial transition pore, resulting in the uncoupling of oxidative phosphorylation and ATP hydrolysis. In addition, the oxidation of voltage-dependent anion channel (VDAC), a mitochondrial outer membrane protein results in the loss of mitochondrial membrane potential (MMP Figure 4). The redox state of vicinal thiol groups in VDAC plays a critical role in the tuning of the voltage sensor of the transition pore and increases its permeability upon oxidation [56]. Moreover, VDAC is present in the outer membrane of mitochondria and is exposed to cytosolic oxidative stress as well. shRNA mediated downregulation of Grx1 results in the oxidation of VDAC but not adenosine nucleotide translocase (ANT), an inner mitochondrial membrane protein. Exposure of Neuro2A cells to β-N-oxalyl amino-L-alanine (L-BOAA), an excitatory amino acid implicated in neurolathyrism also leads to MMP loss, which is alleviated by overexpression of Grx1 [57]. MMP loss results in cristae unfolding in mitochondria facilitating the release of apoptotic factor cytochrome c leading to cell death [58]. Thus, Grx1 functions to maintain mitochondrial integrity during oxidative stress and the downregulation of Grx1 results in mitochondrial dysfunction through oxidative modification of the thiol group present in the outer membrane protein VDAC.

Overall, glutaredoxin acts as a potential neuroprotective mediator to maintain mitochondrial function during oxidative stress, excitatory amino acid toxicity and MPTP mediated neurotoxicity. The role of glutaredoxins will be discussed under each of the disease conditions that follow.

### 1.4. Excitotoxicity and Glutaredoxin

Neurolathyrism is a human neurological disorder caused by the ingestion of plant toxin, 3-oxalylamino-L-alanine (L-BOAA) present in Lathyrus sativus. Neurolathyrism affects motor neurons, and anterior horn cells and results in the loss of axons in the pyramidal tract of the lumbar spinal cord in humans. It has been more commonly seen in men, while women are protected from the disease [59].

Animal models of L-BOAA toxicity showed GSH depletion and loss of mitochondrial complex Ⅰ activity [60] that can be restored by thiol reducing agent, dithiothreitol. GSH depletion has a direct effect on oxidative phosphorylation where it inhibits the complex Ⅰ and reduces ATP production [61]. Upregulation of glutaredoxin mRNA and activation of the AP1 transcription factor is observed within a short time after the systemic administration of L-BOAA. The AP1 transcription factor binding site is present upstream of Grx1 gene and mediates the transcription upon insult through the activation of Jun N-terminal kinase (JNK). Grx1 is critical for the recovery of complex Ⅰ in the motor cortex after excitotoxic insult [62]. Systemic administration of L-BOAA results in the phosphorylation and translocation of cJun to the nucleus, accompanied by activation of AP1 and subsequent increase in glutaredoxin transcription. Overexpression of glutaredoxin could protect against mitochondrial dysfunction during excitotoxicity in motor neurons [63].

A gender specific effect of L-BOAA is seen in human as well as in animal models. L-BOAA mediated GSH depletion and mitochondrial dysregulation are identified only in male [61] and ovariectomized female mice. Higher levels of glutaredoxin are seen in the lumbosacral cord, striatum and the mid-brain regions of female mice which reduces upon ovariectomy, indicating estrogen regulation of Grx1 expression. Ovariectomy downregulates the glutaredoxin expression, GSH levels and complex Ⅰ activity, rendering the female mice vulnerable to L-BOAA toxicity. Treatment of SH-SY5Y cells with estrogen upregulated Grx1 levels and protects from L-BOAA-mediated mitochondrial membrane potential loss and cell death. Therefore, estrogen-mediated Grx1 could probably protect brain regions against excitatory aminoacid toxicity exerted by L-BOAA [63].

### 1.5. Ischemic Reperfusion Injury

GSH homeostasis in the brain during cerebral ischemia has been studied. Reactive oxygen species formed during reperfusion injury following ischemia cause damage to the brain which generally occurs in conditions such as stroke, head trauma and cardiac arrest in humans [64]. Reperfusion following ischemia is detrimental, and causes maximum damage to tissues, in part due to oxidative stress. A major consequence of reperfusion damage is the depletion of GSH accompanied by an increase in free radicals and lipid peroxidation, indicative of oxidative stress.

Reperfusion following severe ischemia, induced by bilateral carotid artery occlusion and systemic hypotension results in massive loss of GSH along with an increased mortality rate [65]. Partial ischemia induced by bilateral carotid artery occlusion for 30 min reduces blood flow to 50%. Total GSH level significantly reduces during reperfusion for 1 h following partial ischemia. Concurrent with the increase in GSSG, there is an increase in malondialdeheyle, indicative of lipid peroxidation. Even though the brain is vulnerable to oxidative stress, it is capable of recovering from moderate oxidative damage, potentially depending on the extent of damage and may vary from region to region. GSH homeostasis or GSH/GSSG ratios are restored after 24 h of reperfusion following partial ischemia [66].

Maintenance of thiol homeostasis is important during reperfusion as it can leads to the oxidation of protein thiols, damage to membrane structures and loss of activity of critical enzymes with active thiol groups such as Na+/K+-ATPase. Thus, the administration of thiol antioxidants such as α-lipoic acid was shown to restore and maintain thiol homeostasis to recover from reperfusion injury. α-lipoic acid shows a remarkable neuroprotective effect during severe ischemic-reperfusion injury with the mortality rate in rats reduced from 78% to 26% accompanied by attenuation of brain GSH loss [65].

### 1.6. Parkinson’s Disease

**Figure 5:**
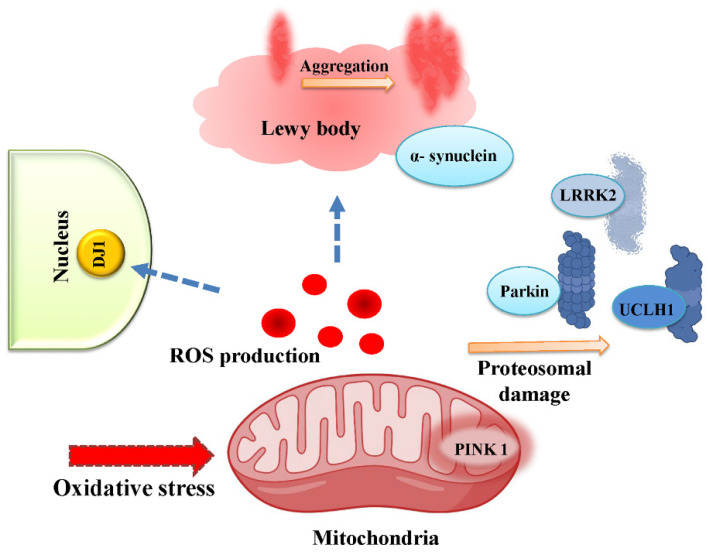
**Familial** PD genes link mitochondrial dysfunction and ROS production to PD pathology. Pink1 is a mitochondrial serine/threonine protein kinase that promotes mitophagy in depolarized mitochondria. PINK1 mutation affects mitochondrial respiration along with DA neuron loss and Lewy pathology. α-synuclein deposition in neurons or mitochondria and loss of parkin function increases oxidative stress, alters mitochondrial membrane potential, complex Ⅰ function and mitochondrial morphology. DJ-1 is involved in response to oxidative stress and may be neuroprotective. Mutations in DJ-1 increase ROS production, accumulation of dysfunctional mitochondria and loss of membrane potential [69]. Mutations in the LRRK2 gene, a common cause of familial and sporadic PD affect mitochondrial dynamics, trafficking and degradation via autophagy [72].

PD is a movement disorder, characterized by the presence of Lewy bodies as intracellular aggregates of α-synuclein and loss of dopaminegic neurons from substantia nigra pars compacta regions (SNpc) [67]. Although the cause of PD is multifactorial, oxidative stress plays a major role in the degeneration of dopaminergic neurons in PD [26]. Several genes identified including parkin, α-synuclein, DJ1, PINK1, and LRRK2, whose mutations are responsible for familial PD, map on to disruption of the ROS scavenging pathway and mitochondrial dysfunctions [68,69,70,71] (Figure 5).

Dopaminergic neurons produce dopamine (DA), a neurotransmitter that modulates synaptic transmission to control motor activity. Dopaminergic neurons are rich in neuromelanin and iron content [73,74]. Both ferrous (Fe^2+^) and ferric states (Fe^3+^) of iron are present in Lewy bodies and iron content is higher in the SNpc regions of the PD brain. When an excess amount of redox-active iron is present in a cell, it generates more ROS, such as superoxides and hydroxyl radicals or lipid peroxides via fenton reaction [75]. The fenton reaction is a chemical reaction between ferrous iron and hydrogen peroxides to form ferric iron and highly reactive hydroxyl radicals (HO•) (Equation (1)). Ferric iron formed reacts with superoxides generated in mitochondria to reduce back to ferrous state (Equation (2)). Hydroxyl radicals are thus formed and react with lipids to form lipid radicals or lipid peroxyl radicals called malondialdehyde (Equation (3)).

(1) $$Fe^{2+}+H_{2}O_{2}\to Fe^{3+}+HO^{-}+HO{•}^{\;}$$

(2) $$Fe^{3+}+O_{2}{}^{•-}\to Fe^{2+}+O_{2}$$

(3) $$HO•+LH(lipids)\to L•(Lipid\;radicals)+H_{2}O^{\;}$$

Iron is mostly associated with proteins in the cell and readily incorporated into mitochondria. Iron-containing enzymes in mitochondria also generates a substantial amount of ROS through a biological process [76,77]. Additionally, DA can be oxidized by iron to produce DA-quinones that can react with the sulfhydryl group of cysteine to modify proteins causing damage to cells and membranes, thus leading to cell death [78,79].

Several animal models are utilized to study different aspects of PD pathology, which include genetic and neurotoxin-induced models. Genetic models, linked to monogenic PD, include a mutation in genes SNCA, LRRK2, UCH-L1, PRKN, and PINK1, and exhibit dopaminergic cell loss, motor deficit and mostly formation of Lewy bodies. Animal models utilizing neurotoxins such as MPTP, rotenone, diamide, and herbicide paraquat, induce loss of dopaminergic neurons and elicit motor symptoms, but mostly lack α-synuclein aggregation seen as Lewy body formation [80].

**Figure 6:**
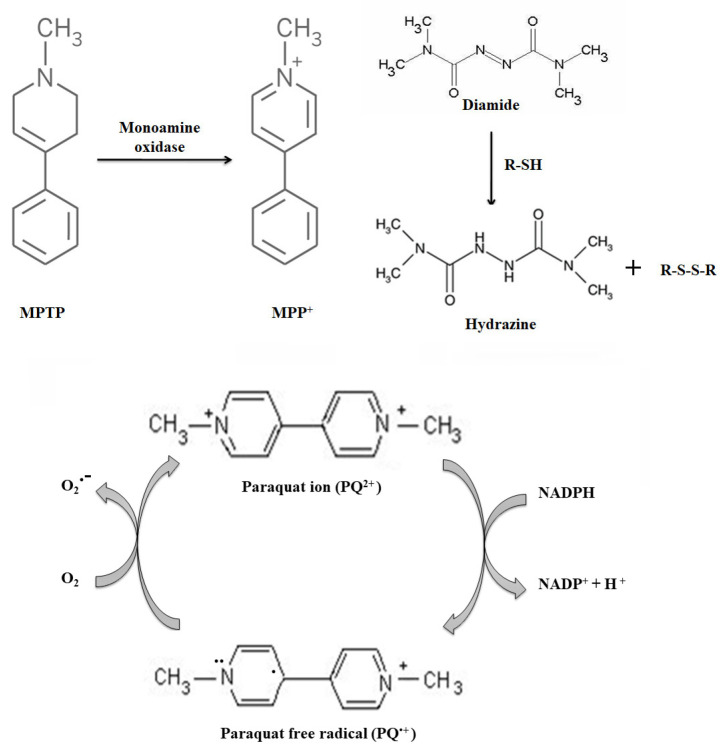
Neurotoxins are used to induce PD phenotype in animal models. MPP^+^ mediates the neurotoxicity of MPTP in astrocytes and neurons. MPTP is converted into MPP^+^ via monoamine oxidase B after crossing the blood-brain barrier. Diamide, an oxidizing agent reacts with protein thiols resulting in the formation of disulfide bond and a hydrazine derivative. Paraquat, a herbicide with a similar structure to MPP^+^, generates ROS by a redox cycling reaction in which the electron transfer to molecular oxygen leading to superoxide production.

MPTP is the most common neurotoxin used to induce parkinsonism to identify early events in PD pathology [81,82]. MPTP increases the number of reactive oxygen species and lipid peroxidation product and is accompanied by a loss in GSH in both the in vitro and the in vivo mouse model. In vivo administration of MPTP results in the lowering of GSH along with increased ROS in midbrain and striatum [83]. MPP^+^, an active metabolite of MPTP (Figure 6) administration inhibits mitochondrial complex Ⅰ activity [84] and is confined to dopaminergic cells which, in part, explains their susceptibility towards oxidative damage in PD [85].

Mitochondrial dysfunction is implicated in PD neurodegeneration [86,87]. Several familial PD genes parkin, PINK1 and DJ1 are linked to mitochondrial pathways (Figure 5). Grx1 is essential for maintaining mitochondrial functions in brain treated with MPTP. They are also involved in the recovery of complex Ⅰ activity after MPTP treatment, which is shown to be impaired with the down regulated Grx1 [50].

In addition to mitochondrial dysfunction, dephosphorylation of Akt1 is seen in MPTP treated animal models. Akt1, a serine/threonine kinase critical for cell survival, undergoes oxidative modification and its impaired activity is observed in PD. Phosphorylated Akt1 mediates cell survival by inhibiting proapoptotic proteins, Bad, caspase and transcription factor FOXO. The oxidation of cysteine residue in Akt1 leads to dephosphorylation, loss of 40% reduced form and inhibition of kinase activity in MPTP treated mice. When cysteine residues at 296 and 310 of Akt1 are oxidized, it increases its association with protein phosphatase2A leading to dephosphorylation [88]. Exposure of cadmium, a pan inhibitor of protein thiol sulfide oxidoreductase or the downregulation of Grx1 in the Neuro2A cell line results in dephosphorylation of Akt1 and decreased Akt1 protein levels. Mutation of critical cysteines at 296 and 310 of Akt1 to serine prevents oxidative modification and increases the cell survival effect of Akt1. MPP^+^ mediated loss in Akt levels is ameliorated by overexpression of Grx1. Therefore, maintaining the thiol status in Akt1 protects it from proteasomal degradation and would be beneficial for cell survival during oxidative damage [89].

Indeed, if Grx1 downregulation causes a similar effect in protein thiol modification as seen with the MPTP model, then a thiol oxidizing agent such as diamide, or diazenedicarboxylic acid bis (N, N-dimethylamide) (Figure 6), would also cause neurodegeneration. One molar equivalent diamide reversibly oxidizes GSH to form a molar equivalent of thiol-disulfide and promotes the conversion of NADPH to NADP+ in less than a minute. Diamide-induced GSH loss persists for a substantial period, unlike transient ischemia and MPTP models which restore thiol homeostasis after an initial insult. Single focal delivery of diamide to substantia nigra targets protein thiols and triggers Parkinsonism phenotypes, such as α-synuclein aggregation, locomotor deficit, and DA neurodegeneration in mice. Disruption of protein thiol homeostasis by diamide triggers neurodegenerative cascade in SNpc neurons and activates the ASK1-p38 MAPK death signaling pathway [90]. Activation of the ASK1-p38 MAPK pathway is a common feature across different PD models. Activated ASK1 mediates the downstream MAPK signaling via p38 MAPK phosphorylation. Downregulating ASK-1 attenuates DA neurodegeneration, indicating its role in neurodegeneration in SN regions [91]. Altogether, maintaining protein thiol homeostasis is important for DA neuron survival as a single administration of diamide causes sustained changes in biochemical and behavioral parameters.

Furthermore, the selective downregulation of Grx1 in SNpc shows a loss of TH positive neurons and the development of motor deficits. A recent study concludes that the knockdown of Grx1 in substantia nigra could selectively degenerate SNpc DA neurons without affecting them in the VTA region. Moreover, significant downregulation of Grx1 mRNA was also observed in the SNpc region of human PD autopsy tissue [92].

It is very well known that typical neuroleptic such as haloperidol, while being a very effective anti-psychotic drug, is known to cause tardive dyskinesia in a subset of patients. Symptoms of dyskinesia overlapping with PD is a very big concern that led to the development of atypical anti-psychotic drugs such as clozapine, quetiapine, etc [93]. Haloperidol induces oxidative stress by increasing the turnover rate of dopamine and shows a similar effect as seen in MPTP in terms of protein thiol oxidation. Acute [94] and chronic [95] administration of haloperidol in rats result in the loss of GSH along with an increase in malondialdehyde, a lipid peroxidation product. A single dose of haloperidol also causes selective inhibition of mitochondrial complex Ⅰ due to the oxidation of essential thiol groups that can be reversed by thiol antioxidant α-lipoic acid [96,97].

Paraquat, a herbicide with structural similarity to MPP^+^, also induces dopaminegic neurodegeneration and mitochondrial dysfunction via oxidative stress, mimicking features of pathological PD [98] (Figure 6). It selectively targets neurons in the SNpc sparing VTA region similar to the observations seen in the PD brain [99,100]. Paraquat generates ROS by redox cycling reaction, in which the herbicide undergoes one-electron reduction to form a paraquat ion followed by an electron transfer to molecular oxygen leading to the formation of superoxides [101] (Figure 6). Paraquat-induced neuronal death is mediated by the phosphorylation of c-Jun N-terminal kinase (JNK) and c-Jun where it activates caspase-3 and apoptosis [102]. Paraquat may inhibit mitochondrial functions through an oxidative mechanism but not through direct inhibition of complex Ⅰ, as in the case of MPTP [98]. Studies conducted to determine the role of Grx1 and protein glutathionylation in paraquat induced nigrostriatal degeneration show a similar effect as seen with MPTP [103]. Indeed, the common features associated with MPTP, diamide, haloperidol, and paraquat, emphasize the selective vulnerability of dopaminergic neurons to oxidative stress and the importance of protein thiol homeostasis.

Several studies on the effect of antioxidant, α-lipoic acid (ALA) across different PD models with a motor deficit and neuroinflammation found promising results in alleviating the symptoms of PD. ALA regulates ferroptosis, a programmed cell death that depends on iron and increased lipid peroxides via activating PI3K/Akt/Nrf2 pathway [104]. ALA reduces the expression levels of inflammatory mediators such as NF-κB, TNF-α, and iNOS, in substantia nigra and improves motor performance by increasing locomotor activity [104].

### 1.7. Alzheimer’s Disease (AD)

Alzheimer’s disease is an age-related progressive form of dementia, characterized by the presence of extracellular amyloid-β deposition, neurofibrillary tangle formations, loss of synapses and neurodegeneration. Multiple pathways contribute to AD pathogenesis and oxidative stress is one of the early events [105,106]. Aβ-42 (Aβ-42) which has a causative role in AD itself produces ROS. Amyloid-β in aqueous solution fragments and generates free radical peptides identified via electron paramagnetic resonance spin trapping. Aβ fragments produce reactive oxygen species and inactivate glutamine synthetase, which is essential for glutamate metabolism [107,108].

Synaptic dysfunction including loss of dendritic spines is one of the major consequences of AD [109] which affects activity dependent protein translation and structural plasticity. Protein translation at the synapse is critical for its maintenance and plasticity. The synthesis of new proteins in response to neuronal stimulation is required for synaptogenesis. The Akt1-mTOR signalling pathway mediates most of the protein translation occurring at the cell and is critical for activity-dependent protein translation. Thiol oxidation of Akt1 due to oxidative stress contributes to synaptic dysfunction in APP/PS1 mice. The oxidation of cysteine groups in Akt1 increases its association with PP2A, causing dephosphorylation and inhibiting its kinase activity. The subsequent loss of Akt1-mTOR signaling affects activity-dependent protein translation at the synapse [110].

Regulating actin dynamics is critical to synaptic functions including synaptic plasticity, memory and learning. The G-actin/F-actin equilibrium is maintained by several actin binding proteins, mainly Cofilin [111]. Cofilin undergoes oxidation in stressed neurons to form dimers and induces the formation of cofilin-actin rods in neurites. Rod formation causes synaptic dysfunctions by disrupting actin dynamics, sequestering dephosphorylated cofilin and increasing mitochondrial membrane potential loss. Cofilin signaling pathways are implicated in synaptic deficits associated with AD. In addition, cofilin-actin rods are observed in cognitively impaired human AD brains [112].

**Figure 7:**
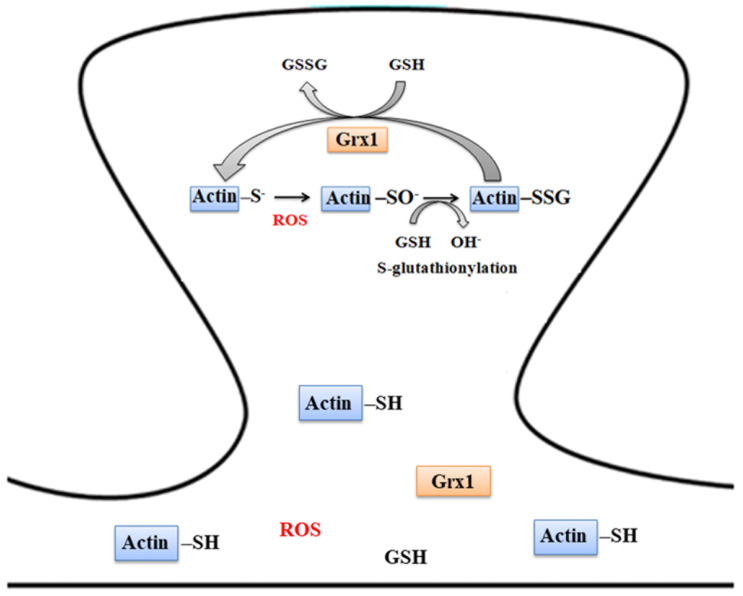
Schematic of actin glutathionylation in dendritic spines. Actin undergoes oxidative modification at the synapse during oxidative stress potentially affecting its assembly and interactions. S-glutathionylation of actin at Cys-374 residue in the presence of GSH affects its polymerization [119]. Actin-SSG formed is reversed effectively by Grx1 utilizing GSH and reducing equivalents of NADPH.

The increased S-glutathionylationin proteins has been identified [113] along with a reduction in thiol oxidoreductase levels in the AD brain compared with age matched controls [114]. Actin, similar to many other proteins, is targets for S-glutathionylation [115,116]. Glutathionylated actin at Cys-374 residue decreases its polymerization rate [117] (Figure 7). A recent study looked at the role of amyloid-β-induced oxidative stress in glutathionylation of actin, showing F-actin loss in the AD mouse model. A reduced form of F-actin is significantly decreased along with an increase in F-actin and G-actin glutathionylation in APP/PS1 mice. Grx1 plays a critical role in maintaining the thiol status of the F-actin cytoskeleton in dendritic spines. The overexpression of Grx1 in the APP/PS1 hippocampus reverses the contextual fear conditioning (cFC) recall deficit, restoring F-actin levels and decreasing ROS levels. Spine morphology and F-actin nanoassembly are restored in primary cortical cultures overexpressed with Grx1. Ameliorating the levels of Grx1 in AD brains could potentially help in maintaining synaptic plasticity and memory deficit [118].

## 2. Conclusions

Maintaining protein thiol homeostasis during oxidative stress is critical in the brain. Over the years, the role of glutathione and glutaredoxin in alleviating oxidative damage has been studied extensively across models of neurodegeneration. Most of the antioxidants do not cross the blood-brain barrier to reverse protein thiol oxidation. Treatment strategies using GSH precursors and common antioxidants such as α-lipoic acid, vitamin E and vitamin C have shown only minimal effects in patients. Hence, it is critical to augment the intrinsic system such as the transcription of glutaredoxins early in the disease progression to effectively combat oxidative stress.

## Data Availability

Not applicable.

## Abbreviations

AD	Alzheimer’s disease
AKt	Intracellular serine/threonine kinase protein kinase B
ALA	α-lipoic acid
ANT	Adenosine nucleotide translocase
AP1	Activator protein 1
APP/PS1	Amyloid Precursor Protein/Presenilin1
ASK	Apoptosis signal-regulating kinase
BSO	Buthionine sulfoximine
cFC	Contextual fear conditioning
Cu, Zn SOD	Copper Zinc superoxide dismutase
Cys	Cysteine
DA	Dopamine
DJ1	Protein Deglycase1
EAAC1	Excitatory amino-acid carrier 1
EAAT	Excitatory amino acid transporter
FOXO	Forkhead box
GPx	Glutathione peroxidase
GR	Glutathione reductase
Grx	Glutaredoxin
GSH	Glutathione
GSSG	Glutathione disulfide
JNK	c-Jun N-terminal kinase
L-BOAA	βeta-oxalylamino-L-alanine
LRRK	Leucine-rich repeat kinase
MMP	Mitochondrial Membrane Potential
Mn-SOD	Manganese-superoxide dismutase
MPP^+^	1-Methyl-4-phenylpyridinium ion
MPTP	1-methyl-4-phenyl-1,2,3,6-tetrahydropyridine
mTOR	Mammalian target of rapamycin
NADPH	Nicotinamide Adenine Dinucleotide Phosphate Hydrogen
NFκB	Nuclear Factor-kappa B
Nrf2	Nuclear factor erythroid 2-related factor 2
P38-MAPK	p38 mitogen-activated protein kinases
PD	Parkinson’s disease
PINK	PTEN-induced putative kinase 1
PrSH	Protein thiol
PrSSG	Protein disulfide
Pr-ss-Pr	Protein mixed disulfides
ROS	Reactive Oxygen Species
SNpc	Substantia Nigra par compacta
TH	Tyrosine hydroxylase
TNF-α	Tumor Necrosis Factor α
VDAC	Voltage-dependent anion-selective channel
VTA	Ventral tegmental area
Xc	Cystine/glutamate antiporter
γ-GCS	γ-glutamylcysteine synthetase
γ-glu	γ-glutamate
